# Molecular Characterization and *In Silico* Analysis of Naturally Occurring TEM Beta-Lactamase Variants among Pathogenic *Enterobacteriaceae* Infecting Indian Patients

**DOI:** 10.1155/2013/783540

**Published:** 2013-10-28

**Authors:** Lena Dhara, Anusri Tripathi, Arijit Pal

**Affiliations:** Department of Biochemistry and Medical Biotechnology, Calcutta School of Tropical Medicine, 108 C. R. Avenue, Kolkata 700073, India

## Abstract

Cephalosporin resistance, particularly due to *bla*_TEM_ encoded *β*-lactamases, among *Enterobacteriaceae* is, though, an increasing public health problem in India; their circulating genetic variants remain unknown. The present study deals with determination of *bla*_TEM_ variants among 134 pathogenic *Enterobacteriaceae* of Indian origin. Their resistance profile against 3rd generation cephalosporins was determined. The presence of *bla*_TEM_ variants among the bacterial plasmids was characterized by PCR followed by sequencing. Intergenic relations among the variants was determined by phylogenetic analysis. *bla*_TEM_ protein were modeled by Modeller9v5 and verified. The catalytic pockets were characterized, and their interaction with cephalosporins was analyzed using AutoDock tools. More than 87% of isolates showed cephalosporin resistance with ESBL production among 57.8% of *Escherichia coli* and 50.6% of *klebsiella pneumoniae*. *bla*_TEM-1_ (84.21%), *bla*_TEM-1_ like (3.94%), *bla*_TEM-33_ (3.94%), *bla*_TEM-116_ (3.94%), *bla*_TEM-169_ (3.94%), and *bla*_TEM-190_ (7.89%) were detected in 76 isolates. Four variants, namely, *bla*_TEM-1_like, *bla*_TEM-33_, *bla*_TEM-169_, and *bla*_TEM-190_, coexisted in 3 isolates. The largest catalytic pocket of *bla*_TEM-33_ explained its expanded activity towards *β*-lactam-*β*-lactamase inhibitor combinations. Molecular docking indicated differential resistance pattern of *bla*_TEM_ variants.

## 1. Introduction

Microbial resistance, especially resistance to cephalosporins, is an ever increasing public health problem worldwide among pathogenic *Enterobacteriaceae, *namely, *Escherichia coli *and* Klebsiella pneumoniae *[1]. Compared to other countries, frequent overuse of antibiotics and crowding of patients with high levels of disease acuity in relatively small, specialized hospital areas in Indian subcontinent have been the selective force to drive this epidemic like antimicrobial resistance development among all major microbial pathogens. Pathogenic bacteria acquire this resistance property due to acquisition of class A *β*-lactamase that hydrolyze *β*-lactam ring of cephalosporins. 

The most commonly encountered class A *β*-lactamase is encoded by plasmid mediated *bla*_TEM_ (Temoniera) gene, which was first isolated from *E. coli *of blood-infected patient [1]. During the catalytic process, this enzyme first uses its active site Ser-70 residue as the key functional group, followed by involvement of Glu-166, Lys-73, Lys-234, and Ser-130 residues to hydrolyze the *β*-lactam ring [2]. However, the substrate binding process is most likely the rate limiting step. So far 204 types of *bla*_TEM_ have been identified that differ at 87 amino acid positions—thus having different protein stability [1]. There is growing biological evidence that increased protein stability (indicated by decrease in Gibbs free energy upon folding, ΔΔ*G*) can lead to protein malfunction and hence diseases [3]. The *bla*_TEM_ variants demonstrate considerable variety with respect to their range of substrate preferences and their levels of hydrolytic activity [1]. Substitutions critical for expanding substrate profile or increasing hydrolytic activities are efficiently selected under selection pressure of *β*-lactam use. During multifocal and multidirectional evolution, two clinically important TEM *β*-lactamases were generated: expanded spectrum TEM (ESBL-TEM) and inhibitor resistant TEM (IRT) *β*-lactamases [4]. ESBL-TEM has expanded substrate spectrum towards oxymino-*β*-lactams, whereas IRT have expanded their substrate profile to the *β*-lactam-*β*-lactamase inhibitor combinations [1].

Despite reports of higher level of 3rd generation cephalosporin resistance and ESBL production in approximately 100% and 87% of pathogenic *Enterobacteriaceae *infecting Indian patients, no study has been done to analyze the involvement of different *bla*_TEM_ variants for development of these properties among pathogenic bacteria [5, 6]. Our earlier study detected 3rd generation cephalosporin resistance among 97% of pathogenic *K. pneumoniae *infecting eastern Indian patients, with presence of *bla*_TEM_ genes among 52% of them [7]. The present study provides molecular insight into the types of *bla*_TEM_ variants circulating among pathogenic *Enterobacteriaceae *of this region, their evolutionary relationship, and their role in development of broad spectrum/inhibitor resistance or ESBL production among these bacteria. This study also attempted to analyze any differences in structure and catalytic activity of these *bla*_TEM_ variants that might have significant implications for development of better antibiotics.

## 2. Materials and Methods

### 2.1. Clinical Isolates

*Enterobacteriaceae *specimens (*n* = 134; *E. coli *= 57, *K. pneumoniae *= 77) were collected from different non duplicate clinical isolates of unrelated patients visiting Calcutta School of Tropical Medicine, Kolkata, India, from June 2011 to October 2012. The bacterial isolates were obtained from patient's urine, blood, throat swab, and wound pus.

### 2.2. Third Generation Cephalosporin Sensitivity, ESBL Production, and MIC Determination

Antibiotic sensitivity and ESBL property of these isolates were determined according to Kirby-Bauer disc diffusion method [8]. The following *β*-lactam antibiotics were used (*μ*g/disc) ceftazidime (30), ceftazidime and clavulanic acid (30/10), cefotaxime (30), cefotaxime and clavulanic acid (30/10), and cefpodoxime (10) (HiMedia Lab Ltd., India). The diameter of zone of inhibition produced by each antibiotic disc was measured, and results were interpreted according to guidelines of Clinical and Laboratory Standards Institute (CLSI). If the zone diameter increased by 5 mm or more, when clavulanate is added compared to the antimicrobial alone, the isolate was considered as ESBL producing. *E. coli *ATCC 352183 was used as positive strain harboring *bla*_TEM_ gene [9]. The minimum inhibitory concentrations (MICs) of ceftazidime and cefotaxime were determined by macrobroth dilution technique, according to CLSI guidelines [10]. Both antibiotics were tested in two-fold dilutions in the range of 16–512 *μ*g/mL.

### 2.3. Bacterial Plasmid Isolation

Plasmid DNA was extracted from the *Enterobacteriaceae *isolates by alkaline lysis method [11]. Briefly, 3 mL overnight culture of patient isolated *Enterobacteriaceae *was lysozyme and RNase treated followed by alkaline lysis, phenol/chloroform extraction, and isopropanol precipitation of the plasmid DNA. Integrity of the plasmids was checked by 1% agarose gel electrophoresis.

### 2.4. Molecular Characterization and Phylogenetic Analysis of *bla*_TEM_ Genes

Amplification of *bla*_TEM_ gene was performed in 96-well thermal cycler (Applied Biosystems, USA) with primers: F: 5′-ATGAGTATTCAACATTTTCGTC-3′; R: 5′-TTACCAATGCTTAATCAGTGAG-3′, generated by using Primer3 (version 0.4.0) [12]. Briefly, each reaction was carried out in 20 *μ*L reaction volume using 1x PCR buffer (Fermentas, USA), 20 pmol of primers (Integrated DNA Technologies, USA), 1 mM of each dNTPs, 1 unit of Taq DNA polymerase (Fermentas, USA), 1.5 mM MgCl_2_, and 100 ng plasmid DNA. Thermocycling parameters were as follows: initial denaturation at 94°C for 60 s, 30 cycles of denaturation at 94°C for 30 s, primer annealing at 55°C for 30 s, and extension at 72°C for 60 s. PCR products were separated on 2% agarose gels and visualized under UV transilluminator (Ultraviolet/Laboratory Products, USA). PCR product of 862 bp size was purified with QIAquick PCR purification kit (Qiagen, USA) and sequenced using BigDye Terminator v3.1 Cycle Sequencing Kit (Applied Biosystems, USA) in 3100-Avant Genetic Analyzer (PE Applied Biosystems Inc, USA). The sequencing was done using both forward and reverse primers for each sample. The nucleotide sequences and deduced protein sequences were analyzed by BLAST and ClustalW programs of the European Bioinformatics Institute (http://www.ebi.ac.uk/) [13]. The sequences were deposited in GenBank database of National Center for Biotechnology Information. Chromatograms were visually inspected for double peaks as signs of presence of different *bla*_TEM_ variants in the same PCR product.

Phylogenetic analysis of our *bla*_TEM_ variants and their evolutionary relationship with those reported in Lahey's mutation database was derived by maximum likelihood method using MEGA software version 5 [14]. The Jones-Taylor-Thornton nucleotide substitution model, selected with the Model Test program according to the Akaike Information Criterion, corrected (AICc), was used as evolutionary model and included a gamma distribution (*G*) with six rate categories and a fraction of invariant (*I*) sites to account for substitution rate heterogeneity among sites.

Amino acid positions in the gene clusters that were presumably subjected to positive selection were identified by application of HyPhy selection test using joint maximum likelihood reconstructions of ancestral states under a Muse-Gaut model of codon substitution and Felsenstein 1981 model of nucleotide substitution as implemented in MEGA version 5. According to the test, codon-by-codon ratio (*ω*) of number of nonsynonymous substitutions per nonsynonymous site (dN) to that of synonymous substitutions per synonymous site (dS), (*ω* = dN/dS) was calculated; values of *ω* > 1 indicated positive selection pressure, whilst values of *ω* < 1 indicated purifying selection pressure, and values of *ω* = 1 represented neutral evolution.

### 2.5. Homology Modeling, Model Validation, and Active Site Identification of *bla*_TEM_ Variants

The PSI-BLAST (Position Specific Iterated-Basic Local Alignment Search Tool) search with default parameters was performed with predicted protein sequences of our *bla*_TEM_ variants against those available in Protein Data Bank (PDB) to find suitable templates for their homology modelling using modelling package MODELLER9v7 [13, 15]. The stereochemical qualities, compatibility of atomic models, and quality factors of these models were verified by PROCHECK, Verify3D, and ERRAT programs of Structural Analysis and Verification Server (SAVES), respectively, and quality of the models was also validated by ProSA server, a web server for Protein Structure Analysis [16].

### 2.6. Variant Stability Prediction

The PyMOL program was used for measuring distance between reactive Ser-70 of the catalytic site and altered amino acid residue [17]. Effect of the alteration on protein stability was predicted by calculating change in free folding energy (ΔΔ*G*) at that site using CUPSAT (Cologne University Protein Stability Analysis Tool) server [18]. The prediction model used amino acid atom potentials and torsion angle distribution to assess the change in solvent accessibility and secondary structure specificity at the mutation site. According to experimental ΔΔ*G* value, each mutation was placed in any of the following three classes: (i) destabilizing mutation: ΔΔ*G* < −0.5 Kcal/mol; (ii) stabilizing mutation: ΔΔ*G* > 0.5 Kcal/mol; (iii) neutral mutation: −0.5 < ΔΔ*G* < 0.5 Kcal/mol [19]. SuperPose (version 1.0) was used to calculate Root-Mean-Square-Deviation (RMSD) values for stability change of *bla*_TEM_ variants compared to wild type (*bla*_TEM-1_), where dissimilarity cutoff >3.0 Å indicated significant change in overall structural stability [20].

### 2.7. Catalytic Pocket Prediction and Measurement

Possible ligand binding pockets containing catalytic residues within the generated models were predicted using Computer Atlas of Surface Topology of Protein (CASTp) server [21]. The catalytic pocket's solvent accessible volume (SV), surface area (SA), and pocket mouth area (MA) were also predicted.

### 2.8. Binding Mode Prediction of *bla*_TEM_ Variants with 3rd Generation Cephalosporins

Mol2 structures of 3rd generation cephalosporins, namely, ceftazidime (ZINC ID: 03830469), cefotaxime (ZINC ID: 04468780), and cefpodoxime (ZINC ID: 14235259), were retrieved from ZINC database [22]. The coordinates of *bla*_TEM_ variants to be used for docking were retrieved using MetaPocket 2.0 server [23]. To study the nature of interactions of *bla*_TEM_ protein models with these cephalosporins, docking was carried out using Autodock 4.0 tools [24]. Energy grid was built within a cubic box of dimensions 60 × 60 × 60 Å grid points and 0.375 Å spacing using the Autogrid program. Docking was performed based on Lamarckian Genetic Algorithm. Grid points were generated around the catalytic pocket to cover the entire ligand binding site, such that the compound to be docked can move freely within it. Docking simulations were performed using Lamarckian Genetic Algorithm (LGA). The docking parameters set to perform each docking experiment were derived from 100 different runs that were set to terminate after a maximum of 2,500,000 energy evaluations, elitism of 1, mutation rate of 0.02, cross-over rate of 0.8, and local search rate of 0.06. The population size was set to 150. Best run coordinates of the docked complex were analyzed and visualized through python molecule viewer and PyMol molecular graphics system for analysis of their mode of interaction with binding site residues. To analyze the molecular interaction between *bla*_TEM_ variants and cephalosporins, complexes were generated using AutoDock tools. LIGPLOT analysis was run for the complexes to understand the hydrogen bonding and hydrophobic interaction within the docked complexes [25].

## 3. Results

### 3.1. Antibiotic Sensitivity Assay and MIC Determination

Total 134 clinical isolates of *Enterobacteriaceae *sp. (*E. coli*, *n* = 57; K. pneumoniae, n* = 77) were collected from different patient sources including urine (*n* = 49; n* = 60), blood (*n* = 1; n* = 9), pus (*n* = 6; n* = 5), and throat swab (*n* = 1; n* = 3). Percentage of resistance towards ceftazidime, cefotaxime, and cefpodoxime among these bacteria was as follows—*E. coli: *92.98% (53/57), 89.47% (51/57), and 100% (57/57); *K. pneumoniae: *89.61% (69/77), 87.01% (67/77), and 100% (77/77), respectively. The ESBL enzyme production was indicated among 57.8% (33/57) of *E. coli *and 50.6% (39/77) of *K. pneumoniae. *MICs of both ceftazidime and cefotaxime were noted to be 64, 128, and 256 *μ*g/mL among 16% (22/134), 59% (79/134), and 25% (33/134) of *E. coli *and 1.5% (2/134), 54% (72/134), and 45% (60/134) of *K. pneumonia,* respectively.

### 3.2. Molecular Characterization of *bla*_TEM_ Variants

**Figure 1:**
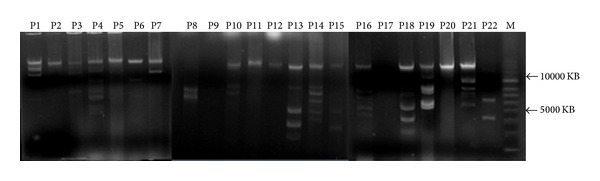
Plasmid profiles of pathogenic *Enterobacteriaceae.* P1-P22: plasmids of pathogenic bacterial isolates; M: supercoiled DNA ladder.

**Figure 2:**
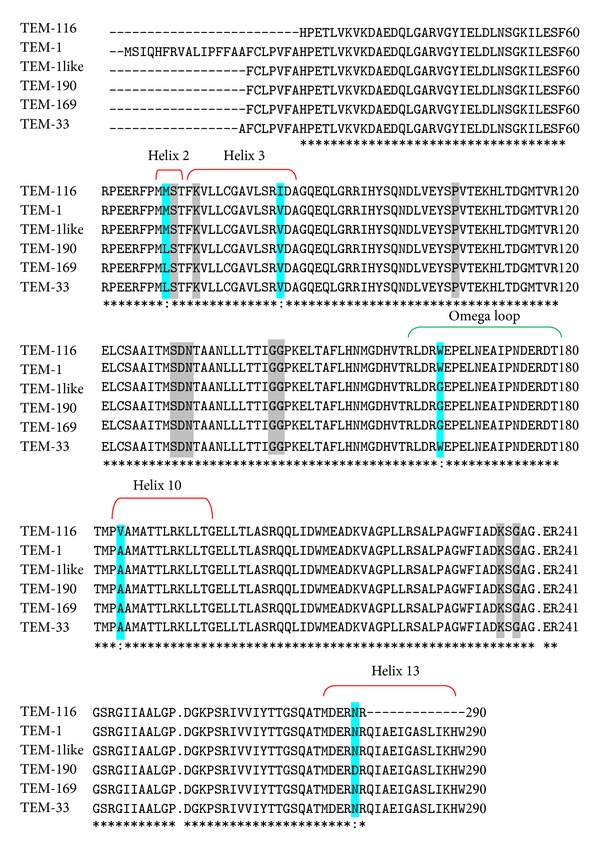
Multiple sequence alignment of *bla*_TEM_ variants detected among *Enterobacteriaceae *infecting Indian patients. Variable regions of the proteins are marked in cyan and the conserved regions of class A *β*- lactamase are marked in gray colour. The amino acid numberings are according to ABL scheme. Asterisks indicated identical amino acids; “.” indicates a postulated deletion; blank spaces indicate one or more residues omitted from the alignment.

Majority of the bacterial isolates harbored more than one plasmid: 2–9 bands were found in 94 isolates, whereas 1 band was found in 40 bacterial samples (Figure 1). The *bla*_TEM_ gene was detected in 60.29% (35/57) of *E. coli* and 60.34% (41/68) of *K. pneumonia, *respectively. Sequencing of PCR amplified *bla*_TEM_ genes (*N* = 76) revealed presence of *bla*_TEM-1_ (84.21%) (*N* = 64, GenBank accession ID: **JN002395**), *bla*_TEM-116_ (3.94%) (*N* = 3, GenBank accession ID: **JF973688**), and *bla*_TEM-190_ (7.89%) (*N* = 6, GenBank accession ID: **KC699842**) (Table 1). Interestingly *bla*_TEM-1_like, *bla*_TEM-33_, *bla*_TEM-169_, and *bla*_TEM-190_ were suggested to coexist in 3 plasmid samples as evidenced by the presence of double peaks C/A (M), G/T (K), and A/G (R) in their discriminatory regions at codons 69, 165, and 276 (GenBank accession ID: **KC699843**). Among them, *bla*_TEM-1_, *bla*_TEM-1_ like, *bla*_TEM-116_, *bla*_TEM-169_, and *bla*_TEM-190_ belonged to broad spectrum group of *bla*_TEM_, whereas *bla*_TEM-33_ was inhibitor resistant *bla*_TEM_. None of the detected *bla*_TEM_ variants were of ESBL type. Amino acid variations among these *bla*_TEM_ variants and their respective positioning in the protein have been depicted in Figure 2. Structurally, these *bla*_TEM_ variants differed at any of the five amino acid positions with respect to *bla*_TEM-1_ (M69L, V84I, W165G, A184V, and N276D). Only W165G was found to be located within the omega loop, whereas other four variations were located within different helical regions of the protein.

**Figure 3:**
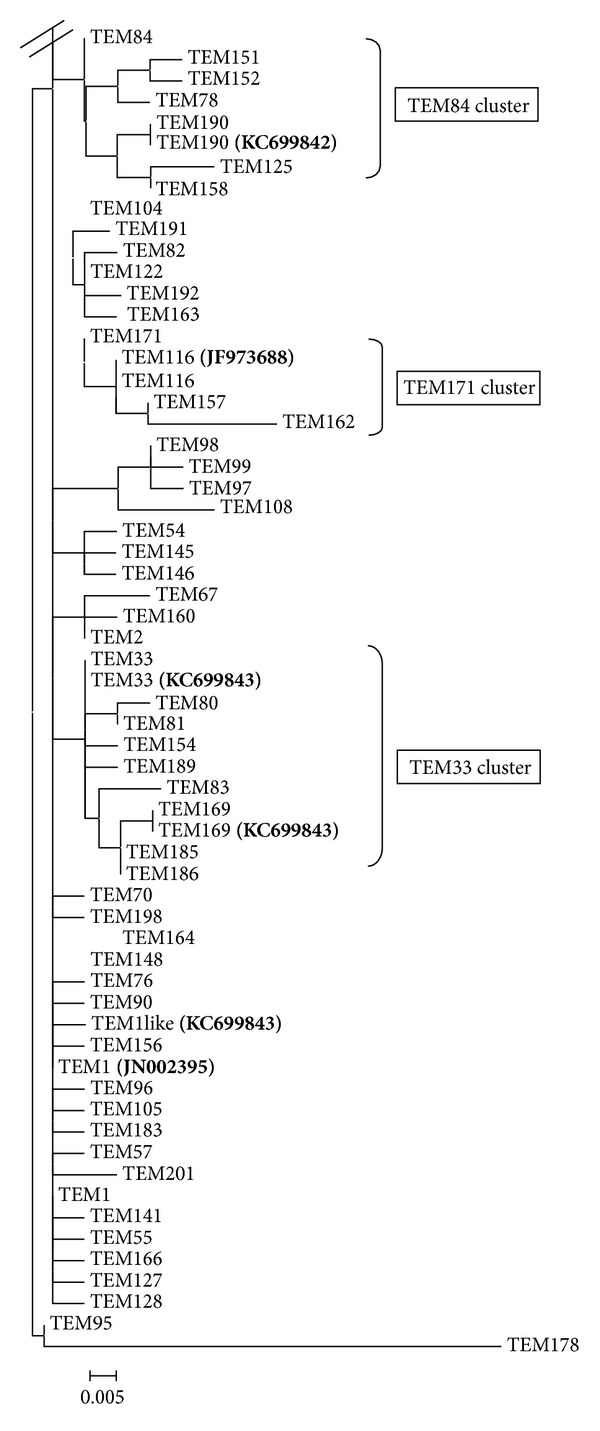
Partial dendrogram of the *bla*_TEM_ variants available in Lahey's database. Clusters harboring our detected *bla*_TEM_ variants are marked in bold.

Maximum likelihood phylogenetic analysis of our *bla*_TEM_ variants along with those reported in Lahey's database generated a dendrogram that showed different *bla*_TEM_ clusters (Figure 3). Among them, *bla*_TEM-190_, *bla*_TEM-116_, *bla*_TEM-33_, and *bla*_TEM-169_ were present within *bla*_TEM-84_, *bla*_TEM-171_, and *bla*_TEM-33_ subclusters, respectively. Analysis of our *bla*_TEM_ sequences along with those available in Lahey's database for detecting any evolutionary selection pressure identified sixteen amino acid positions (39, 104, 164, 165, 182, 196, 238, 240, 244, 265, 268, 275, 276, 278, 288, and 289) which were under positive pressure (*ω* > 1). Among them, amino acids at 165 and 276 positions, with *ω* values of 2.54118 and 2.27695, respectively, varied among our *bla*_TEM-1_ like, *bla*_TEM-169_, and *bla*_TEM-190_.

### 3.3. Homology Modelling and Validation of bla_TEM_ Proteins

The 3D model of proteins provided invaluable insights into the structural basis of its function. Search of PDB confirmed presence of templates for each of our *bla*_TEM_ variants; namely, protein with PDB ID: 1AXB (at resolution 2.00 Å) was selected for *bla*_TEM-116_ and that with PDB ID: 1ZG4 (at resolution 1.55 Å) was selected for rest of the *bla*_TEM_ variants. Our *bla*_TEM_ variants showed 93-94% sequence identity with the selected templates (Table 2). Ramachandran plots confirmed good stereochemical quality of the models—as evidenced by the number of residues in most favoured, additional allowed and generously allowed regions, respectively, and no residue in the disallowed region. The overall quality factors of 3D models predicted by ERRAT were above 94.553 in all the models, except for *bla*_TEM-1_ with ERRAT score of 80.292. Verify 3D server predicted that 80% of the residues of our models had an average 3D–1D, so the models were also verified by Verify 3D. *Z*-score values indicated that the input structures were within the range typically found for similar sized native proteins.

### 3.4. *bla*_TEM_ Variant Stability

The distance between Ser-70 of the catalytic site and altered/wild type residue at position 69 was M/L = 2.5 Å, at position 84 was V/I = 22.0 Å, at position 165 was W/G = 10.9 Å, at position 184 was A/V = 17.4/17.5 Å, and at position 276 was N/D = 15.4 Å. Overall stability change, calculated using both atom potential and torsion angle distribution, was found to increase due to four alterations (W165G, M69L, A184V and N276D) and decrease due to V84I change (Table 3). The RMSD (*α* carbon) values of *bla*_TEM_ variants with respect to *bla*_TEM-1_ (wild type) were 2.24 Å (*bla*_TEM-1_ like), 0.43 Å (*bla*_TEM-116_), 2.58 Å (*bla*_TEM-169_), 2.46 Å (*bla*_TEM-190_), and 3.02 Å (*bla*_TEM-33_).

### 3.5. Measurements of Catalytic Pockets and Active Binding Site Analysis

The SV, SA, and MA of the catalytic pockets varied among our *bla*_TEM_ variants, in the following order: *bla*_TEM-33_ > *bla*_TEM-116_ > *bla*_TEM-1_ > *bla*_TEM-1_ like > *bla*_TEM-169_ > *bla*_TEM-190_ (Table 2). Approximately 12 times increase in SV, 9.5-fold increase in SA, and 3-fold increase in MA were noticed in inhibitor resistant *bla*_TEM-33_ compared to that of *bla*_TEM-190_, one of the broad spectrum variants. The most potential catalytic residue containing ligand binding pocket of *bla*_TEM_ variants was also validated by metaPocket 2.0.

### 3.6. Binding Mode Analysis of *bla*_TEM_ Variants with Ceftazidime, Cefotaxime, and Cefpodoxime

**Figure 4:**
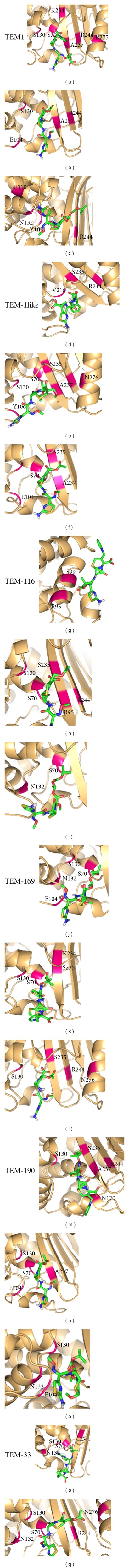
Binding mode analysis of 3rd generation cephalosporins with *bla*_TEM_ variants in the docked complexes. Left panel shows the molecular interaction of docked *bla*_TEM_ variants with ceftazidime ((a), (d), (g), (j), (m), and (p)), middle panel shows molecular interaction with cefotaxime ((b), (e), (h), (k), (n), and (q)), and right panel shows interaction with cefpodoxime ((c), (f), (i), (l), and (o)); *bla*_TEM_ variants are represented as light orange cartoons; the three antibiotics are represented by stick, and the interacting amino acids are represented in pink colour.

The negative low docking energy (Δ*G* ^0^ value) of docked complexes indicated strong favorable interaction between *bla*_TEM_ variants and 3rd generation cephalosporins, whereas low inhibition constant (K_m_ value) indicated high affinity of *β*-lactamase for these antimicrobials (Table 2, Figure 4). Considering Δ*G* ^0^ and K_m_ values of cephalosporin-*β*-lactamase interaction, two patterns were observed among the detected *bla*_TEM_ variants. The *bla*_TEM-1_ like, *bla*_TEM-116_, *bla*_TEM-190_, and *bla*_TEM-33_ (Group I) might interact with cephalosporins in the following order: cefotaxime > ceftazidime > cefpodoxime, whereas *bla*_TEM-1_ and *bla*_TEM-169_ (Group II) might interact with cephalosporins in the order: ceftazidime > cefotaxime > cefpodoxime. However, for both Groups I & II *bla*_TEM_s, distance between carbonyl carbon atom of *β*-lactam ring of cephalosporin and Ser-70 of *bla*_TEM_ was lowest for cefotaxime.

## 4. Discussion

Our study indicated high degree of 3rd generation cephalosporin resistance among pathogenic *Enterobacteriaceae *infecting Indian patients. The most common cause of cephalosporin resistance is acquisition of plasmid encoded *bla*_TEM_ gene variants that produce *β*-lactamases with altered degree of cleaving capacity towards 3rd generation cephalosporins. As multiple plasmids were found in majority of our bacterial isolates, higher chance of *β*-lactamase gene mobilization existed in eastern Indian patient population indicating higher bacterial clonality for *bla*_TEM_ carriage.

The present study is the first to report presence of six *bla*_TEM_ variants, namely, *bla*_TEM-1_, *bla*_TEM-1_ like, *bla*_TEM-116_, *bla*_TEM-169_, *bla*_TEM-190_, and *bla*_TEM-33_, in patient-isolated *E. coli and K. pneumoniae *from India. Multiple *bla*_TEM_ variants were suggested to coexist among 3 clinical isolates indicating extensive role of TEM *β*-lactamases for resistance against cephalosporins in our patient isolates. Interestingly, our isolated *bla*_TEM_s encoded five types of broad spectrum *β*-lactamases and one inhibitor resistant TEM *β*-lactamase. Though ESBL production was indicated in more than 50% of the analyzed bacteria, no ESBL type TEM was found in our study indicating limited role of TEM *β*-lactamases and presence of other ESBL genes within them, thus, imparting bacterial ESBL property. This is the first report from India to detect presence of multiple *bla*_TEM_ variants, as only *bla*_TEM-1_ has been previously reported from southern India [26]. *bla*_TEM-1_ like variant has not been previously reported elsewhere. Selection pressure at 165th and 276th residues of our *bla*_TEM-1_ like, *bla*_TEM-169_ and *bla*_TEM-190_ proteins indicated positive selection pressures during natural evolution of these variants.

These *bla*_TEM_ variants differed at five amino acid positions: M69L, V84I, W165G, A184V, and N276D. In case of M69L, distance measurement of M/L at position 69 from Ser-70 indicated no significant structural change due to this alteration. Moreover, both Met and Leu were good helix-forming residues, and ΔΔ*G* value of M69L conversion indicated it to be a stabilizing mutation [27]. In case of V84I, both *β*-branched Val and Ile at position 84 had more bulkiness near the protein backbone, which might restrict the protein to adopt an alpha- helical conformation [28]. Also, significant change in solvent accessibility and negative ΔΔ*G* value of V84I conversion indicated it to be less stabilizing mutation. Among all the substitutions, only W165G was located within the omega loop. Both Trp and Gly were nonpolar amino acids; hence, this conversion did not change the protein polarity. But due to side-chainless property of Gly, Gly-containing *bla*_TEM_s might have much more conformational flexibility which might play an important role to maintain the omega loop structure [27]. Earlier studies also reported that flexibility of the omega loop might allow the distance between two residues to be shortened during the acylation process of catalytic mechanism [29]. No change in distance of Ser-70 from Trp/Gly at position 165, very little change in solvent accessibility, and positive ΔΔ*G* value of W165G indicated that no significant structural change was caused due to this substitution (Table 3). In case of A184V, Ala, one of the best helix-forming residues, was replaced by *β*-branched Val, which was invariably a poor helix former and for this conversion on *α*-helix-10, overall stability was decreased [27, 28]. There was significant change in solvent accessibility due to A184V substitution, with Val-184 of the helix being totally exposed on the enzyme surface.

Solvent accessible pocket volume/area of these six types of *bla*_TEM_s indicated differential antibiotic spectrum among them (Table 2). Greatest SV, SA, and MA of *bla*_TEM-33_, compared to other *bla*_TEM_ variants, might explain its expanded substrate profile towards *β*-lactam–*β*-lactamase inhibitor combinations. RMSD value of 3.02 Å of *bla*_TEM-33_, compared to *bla*_TEM-1_, referred to significant change in its structural stability. Previous studies reported that minimum free energy of interaction or tight binding for an enzyme-antimicrobial complex was regarded as an indicator of resistance against antimicrobials [29]. Δ*G* ^0^ and K_m_ values of the docked complexes indicated that for Group I *bla*_TEM_ variants, cefotaxime might interact more efficiently than ceftazidime/cefpodoxime—thus imparting greater resistance towards cefotaxime than other two antibiotics; whereas in case of Group II *bla*_TEM_ variants, ceftazidime might interact more efficiently than cefotaxime/cefpodoxime—thus showing higher ceftazidime resistance. However, resistance pattern of the analyzed bacteria towards 3rd generation cephalosporins could not be explained solely due to presence of *bla*_TEM_ groups, as other *β*-lactamase encoding genes were found to be present within them (data not shown). Hydrogen bonds and hydrophobic interaction played a critical role in stabilizing protein-ligand complexes and therefore contributed significantly to improving binding affinity and efficacy of the antimicrobial [30]. LIGPLOT analysis that revealed catalytic residues Ser-70 and Ser-130 might play critical role in stabilizing the docked complexes by hydrogen bonding (Table 2). *bla*_TEM-1_ and *bla*_TEM-169_ were also stabilized by K234, another catalytic residue. Amino acids R244, S235, A237, and N132 are frequently hydrogen bonded with the antibiotics indicating their crucial role during molecular interactions. An increase in number of hydrophobic atoms in the active core of antimicrobial-target interface further increases the binding affinity between protein-antimicrobial interfaces. In case of Group II *bla*_TEM_ variants, the number of amino acids showing hydrophobic interaction with ceftazidime was higher than that with cefotaxime—thus validating greater resistance of these variants towards ceftazidime. Similarly, in case of *bla*_TEM-116_ and *bla*_TEM-1_ like of Group I, a higher number of hydrophobic interactions were found in case of cefotaxime than ceftazidime—thus indicating their greater resistance property towards cefotaxime. However, *bla*_TEM-190_ and *bla*_TEM-33_ did not follow this pattern.

## 5. Conclusion

Thus, the study indicated significant role of multiple TEM *β*-lactamases in imparting 3rd generation cephalosporin resistance among pathogenic bacteria infecting Indian patient population. *In silico *analysis predicted differential antibiotic resistance pattern among *bla*_TEM_ variants. Hence, early detection of antibiotic resistant gene variants could guide the choice of optimal antibiotic therapy for successful treatment—thus improving the outcomes for patients with severe *Enterobacterial *infections.

## Figures and Tables

**Table 1:** Characterization of *bla*_TEM_ variants identified in this study.

TEM types	Matched GenBank accession ID	Our accession ID	Enzyme type			Codon alterations compared to TEM-1					
			Broad spectrum	ESBL	IRT	69	84	134	165	184	276
TEM-1	YP001928075	JN002395	**+**	−	−	ATG	GTT	GCT	TGG	GTC	AAT
TEM-116	AEJ08195	JF973688	**+**	−	−	CTG	ATT	−	−	GCC	−
TEM-1like	ACP18864	KC699843	**+**	−	−	−	−	−	GGG	−	−
TEM-169	ACP18864	KC699843	**+**	−	−	CTG	−	−	GGG	−	−
TEM-190	AEL88240	KC699842	**+**	−	−	CTG	−	−	GGG	−	GAT
TEM-33	AF190695	KC699843	−	−	**+**	CTG	−	GCG	GGG	−	−

**Table 2:** Model validation of *bla*_TEM_ variants and docking details of these variants with 3rd generation cephalosporins.

TEM variants	PDB ID of the templates (% sequence identity)	SAVES & ProSA result of protein validation		CASTp result of pockets (solvent accessible)			Antibiotics	Distance	Docking Energy (Kcal/mol)	Inhibition constant Ki (mM)	H-bonding residues	Residues involved in making hydrophobic contacts
		*Z*-scores, ERRAT, and Verify3D	PROCHECK	Volume	Area	Area of pocket						
TEM-1	1ZG4 (94%)	*Z*-scores: −7.46 ERRAT: 80.292 Verify3D: 80%	*X* = 94.0% *Y* = 4.3% *Z* = 1.7%	50.8	64.6	28.4	CAZ	2.6	−4.69	0.36	S70, S130, K234, A237, R244, and R275	Y105, N170, V216, E222, S235, G238, and M272
							CTX	2.5	−4.59	0.43	E94, S130, A237, and R244	S70, Y105, N170, and G236
							CEP	9.1	−3.98	1.21	Y105, E110, N132, and R244	S70, A237

TEM-116	1AXB (94%)	*Z*-scores: −6.93 ERRAT: 94.553 Verify3D: 80%	*X* = 92.1% *Y* = 7.0% *Z* = 0.9%	70.4	93.1	36.3	CAZ	17.5	−4.30	0.70	R95, S99	A188, K190
							CTX	2.7	−5.37	0.12	S70, S130, S235, A237, and R244	Y105, N170, G236, and E240
							CEP	8.1	−3.45	2.96	S70, N132	E104, Y105, P107, S130, P167, N170, S235, G236, and A237

TEM-1ike	1ZG4 (94%)	*Z*-scores: −6.93 ERRAT: 94.553 Verify3D: 80%	*X* = 95.7% *Y* = 2.6% *Z* = 1.3% *Z*′ = 0.4%	27.5	44.0	24.5	CAZ	8.4	−4.29	0.72	V216, S235, and R244	Y105, S130, K215, and A237
							CTX	2.5	−4.57	0.44	E104, R244, S235, S70, and A237	Y105, V216, G236, G238, E240, and M272
							CEP	6.8	−3.13	5.10	S70, Y107, S130, S235, A237, and R244	V216, G236, and A270

TEM-169	1ZG4 (94%)	*Z*-scores: −6.93 ERRAT: 94.553 Verify3D: 80%	*X* = 94.8% *Y* = 3.9% *Z* = 1.3%	19.3	31.1	18.5	CAZ	9.7	−4.60	0.42	S70, S130, K234, and S235	E104, Y105, N132, P167, N170, V216, A237, and E240
							CTX	3.9	−4.53	0.48	S130, S235, R244, and N276	Y105, N170, V216, and A270
							CEP	7.5	−3.16	4.83	S70, E104, S130, and N132	Y105, N170, and A237

TEM-190	1ZG4 (94%)	*Z*-scores: −8.57 ERRAT: 95.686 Verify3D: 80%	*X* = 94.0% *Y* = 4.3% *Z* = 1.7%	6.6	10.3	14.3	CAZ	9.8	−3.59	2.30	S70, S130, S235, A237, and R244	Y105, N132, P167, E168, E171, V216, and E240
							CTX	2.4	−3.65	2.10	S70, E104, S130, and A237	Y105, N170, V216, G238, and E240
							CEP	7.2	−2.83	8.43	S70, E104, S130, and N132	Y105, E166, P167, and N170

TEM-33	1ZG4 (94%)	*Z*-scores: −6.93 ERRAT: 94.553 Verify3D: 80%	*X* = 94.9% *Y* = 3.4% *Z* = 1.7%	81.3	97.8	44.5	CAZ	9.8	−4.48	0.52	S70, S130, N132, and K234	Y95, N168, V216, S235, G236, A237, and E240
							CTX	3.1	−5.19	0.15	S70, S130, N132, R244, and N276	Y95, N168, P219, and S235
							CEP	9.5	−3.15	4.95	—	E94,Y95, P97, N132, V216, and S235

**Table 3:** *Bla*_ TEM_ protein stability analysis at five differentiating amino acid positions.

Amino acid substitution	Change in solvent accessibility (%)	Free folding energy change (ΔΔ*G*) (kcal/mol)
Met69Leu	1	2.0
Leu69Met	0.55	−3.82

Val84Ile	24.72	−0.48
Ile84Val	15.14	−1.86

Trp165Gly	37.84	0.9
Gly165Trp	34.31	−1.61

Ala184Val	27.22	0.59
Val184Ala	15.61	−1.25

Asn276Asp	8.2	0.09
Asp276Asn	9.72	−1.59
